# The relationship between psychological capital, stress, micro-learning environment, and professional identity in nursing interns: a structural equation modeling study

**DOI:** 10.3389/fpsyg.2025.1458384

**Published:** 2025-03-17

**Authors:** Boxiong Gong, Xin Chen, Na Wang, Yan Zhan, Huiqin Zhong, Rong Zhang, Yi Luo, Zhentong Zhang, Longti Li

**Affiliations:** ^1^Innovation Centre of Nursing Research, TaiHe Hospital, Hubei University of Medicine, Shiyan, Hubei, China; ^2^Nursing Department, TaiHe Hospital, Hubei University of Medicine, Shiyan, Hubei, China; ^3^School of Nursing, Ningbo College of Health Sciences, Ningbo, Zhejiang, China

**Keywords:** professional identity, psychological capital, stress, learning environment, nursing intern

## Abstract

**Background:**

Nursing interns play a crucial role in shaping the future nursing workforce, as their professional identity is closely linked to workplace retention rates and patient outcomes. Thus, investigating the factors that influence nursing interns' professional identity is important.

**Objective:**

To evaluate the relationship between psychological capital, stress, micro-learning environment, and professional identity among nursing interns.

**Methods:**

This was a cross-sectional study. The sample consisted of 388 nursing interns from 8 comprehensive teaching hospitals across five cities in Hubei Province between March and April 2024. Data were collected using a Descriptive Information Questionnaire, the Psychological Capital Questionnaire, the Student Nurse Stress Index scale, the Healthcare Education Micro Learning Environment Measure, and the Professional Identity Scale. The structural equation model was applied to explore the influencing factors of nursing interns' professional identity.

**Results:**

The mean total score for professional identity was 35.57 ± 7.47. Psychological capital positively influenced professional identity directly (β = 0.16, *P* < 0.01), while stress as measured using the student nurse stress index had a negative impact (β = −0.20, *P* < 0.01). High scores on the healthcare education micro-learning environment measure positively contributed to the development of professional identity (β = 0.69, *P* < 0.001). Furthermore, psychological capital was identified as a mediator in the association between the healthcare education micro learning environment and professional identity, as well as between the student nurse stress index scores and professional identity.

**Conclusion:**

The study suggests that a higher level of psychological capital, low stress levels, and a satisfied micro-learning environment are essential in fostering professional identity among nursing interns. It recommends collaboration between nursing schools and clinical departments to enhance nursing interns' psychological capital and stress management skills, creating a positive and safe working environment, thereby fostering professional identity among nursing interns.

## 1 Introduction

Nurses are essential to the delivery of healthcare, yet high turnover rates and shortages pose a universal challenge (WHO, 2020). It is estimated that by 2030, all countries must increase the annual number of nurse graduates by an average of 8% to mitigate the shortage and improve their capacity to employ and retain these graduates (WHO, 2020). Research shows that turnover rates among new nurses in their first year range from 8 to 69% (WHO, 2020; Zhang et al., 2019), adding to nursing shortages and financial losses. Nursing students play an important role in shaping the future nursing workforce, as their perceptions, behaviors, and attitudes toward professionalism significantly influence their career decisions and the cohesion of the nursing team (Lin et al., 2024). Studies indicate that certain individuals entering the nursing field may lack the aspiration to pursue nursing as a career or possess an incomplete understanding of nursing accountability (Lin et al., 2024). Clinical nursing internships represent a critical stage in professional identity formation (Lin et al., 2024; Yao et al., 2021), this is the stage when nursing interns apply theoretical knowledge to clinical practice which facilitates the transformation of their identity from nursing students into professional nurses. However, nursing interns may experience various pressures due to their inadequate skills, unfamiliarity with clinical practice, potential danger of healthcare settings and the fierce competition for nursing promotion (Lin et al., 2024; Ugwu et al., 2023; Yang et al., 2023). Therefore, promoting the formation of professional identity among nursing interns is essential for addressing nurse shortages and fostering a cohesive nursing team.

Professional identity in nursing refers to “a sense of oneself, and about others, influenced by the characteristics, norms, and values of the nursing discipline, leading an individual to think, act, and feel like a nurse” (Giddens, 2021). Professional identity plays a vital role in shaping the landscape of nursing education and practice. It not only directly influences nursing students' retention but also consistently predicts the intention to leave among novice nurses (Kelly et al., 2017; Zhang et al., 2017). A stronger professional identity is essential for reducing stress, boosting job satisfaction, self-confidence, decision-making abilities, clinical performance, feelings of achievement and workplace retention rates and for fostering robust inter-professional relationships, facilitating the delivery of high-quality care which improves patient outcomes (Zhao et al., 2021; Fitzgerald, 2020; Tao et al., 2023; Yao et al., 2021; Zhong et al., 2024). Conversely, a weaker professional identity is linked to moral distress, values dissonance, and the risk of external definitions shaping the nursing profession (Fitzgerald, 2020; Giddens, 2021; Sang et al., 2022; Zhong et al., 2024). However, nursing students' professional identity scores vary globally, with the majority remaining at low to moderate levels (Sang et al., 2022; Wei et al., 2021; Zeng et al., 2022). Nursing interns' professional identity will directly influence their patient safety attitudes (Yang et al., 2023). Recognizing the significance of professional identity in nursing interns, it is critical to identify those who exhibit low levels and employ effective approaches to enhance their professional wellness. These influencing factors include age, level of education, major selection, residency status, workplace environment, clinical learning experience, teaching quality, vocational planning, and related information on social media (Li et al., 2022; Wei et al., 2021; Wu et al., 2020; Zeng et al., 2022). Furthermore, nursing interns confronted with workplace violence or adverse events may experience a reduction in their professional identity (Yang et al., 2023). Adequate instructor support and fostering positive hospital relationships are crucial for enhancing nursing interns' professional identity (Yang et al., 2023). However, in existing studies a scoping review reveals the absence of confirmed findings regarding the associations between nursing students' professional identity levels and their demographic characteristics (Liu et al., 2023). Based on the Social Cognitive Career Theory (SCCT) (Lent et al., 1994), career-related outcomes, such as professional identity, are shaped by the dynamic interaction of cognitive variables (e.g., PsyCap), supportive environmental factors (e.g., micro-learning environments), external challenges (e.g., stress), and individual characteristics. Nevertheless, there is limited understanding regarding the interplay of PsyCap, stress, micro learning environment, and professional identity among nursing interns.

PsyCap, rooted in positive psychology and positive organizational behavior, is formally described as “a positive psychological state demonstrated by individuals as they grow and develop” (Luthans et al., 2004), encompassing four aspects: self-efficacy, hope, resilience, and optimism (Luthans et al., 2006). Hope is finding alternative ways to achieve goals; optimism is maintaining a positive view of the expected outcomes; resilience is the capacity to recover after adverse events; and efficacy is the confidence one has during the completion of a challenging task (Luthans et al., 2015). Widely studied in nursing, PsyCap reflects positive mental states and closely relates to wellbeing and managing workplace stress (Mubarak et al., 2021). Enhanced levels of PsyCap correlate with desirable work-related outcomes (Flinkman et al., 2023), while lower levels may worsen negative emotions like stress, compassion fatigue and burnout, leading to increased turnover intentions (Elliott and Fry, 2021; Flinkman et al., 2023; Labrague et al., 2020; Liu et al., 2021; Ren et al., 2021; Xiao et al., 2022). Furthermore, PsyCap could contribute to mediating the connection between workplace violence and professional identity among doctors and psychiatric nurses (Chang et al., 2023; Qiu et al., 2019). A study on medical interns suggests that PsyCap mediates significantly between job stress and professional identity (Liu et al., 2023). In nursing students, PsyCap mediates between clinical work stress and anxiety among master's degree candidates and also influences perceived social support and bullying behavior among nursing students in their education (Ding et al., 2024; Ling and Yu, 2023). While several research investigations have highlighted the importance of PsyCap in nurses and medical interns, the connection between PsyCap and professional identity among nursing interns has not yet been explored.

Stress is characterized as the physiological or psychological reaction to internal or external stressors (American Psychological Association, 2018), and can have varied effects on individuals. Positive stress supports professional growth by enhancing learning, skills, ethics, communication and academic performance (Araújo et al., 2023). Conversely, negative stress, with physical, psychological, and behavioral symptoms like headaches and anxiety, can lead to depression, suicide, program dropout, career uncertainty, identity issues, decreased motivation, and declining academic performance (Araújo et al., 2023). Stress is a dynamic interaction between an individual and their environment. Transitioning from classroom learning to clinical practice poses significant stress and challenges for many nursing interns, who often find it difficult to adapt to the professional environment (Ugwu et al., 2023). Nursing interns' stress levels were primarily moderate (Zheng et al., 2022). Evidence suggests that nursing students' resilience and professional identity have a significant impact on their stress levels, revealing an inverse relationship between stress and professional identity (Zhao et al., 2021). Research shows that stress impacts the professional identity in medical interns, mediated by psychological capital (Liu et al., 2023). However, a substantial research gap persists regarding nursing interns and their experiences during their internship periods.

The learning environment is a dynamic social structure where students engage with patients, supervisors, peers, and staff to acquire knowledge (Hasnain et al., 2024). The clinical learning environment significantly impacts nursing interns' resilience level and academic achievement. It fosters the development of clinical skills, patient safety behavior, problem-solving abilities, and clinical reasoning, with supervision quality enhancing skills and averting burnout through support from peers, seniors, and staff (Huang et al., 2024; Rodriguez-Garcia et al., 2021; Xu et al., 2024). Furthermore, the environment directly influences nursing interns' professional identity and also indirectly influences it through ego identity (Xia et al., 2023). Comprehending the impact of the environment on nursing intern learning can pose challenges due to its multifaceted nature, influenced by different organizational levels (Isba et al., 2020). The learning environment comprises various micro learning environments, and is dynamic. A micro learning environment is a smaller-scale entity that collectively makes up and influences the overall learning environment, two essential micro learning environments encompass the staff's attitude and behavior toward students, as well as the teaching and supervision quality during clinical placements (Isba et al., 2020). Investigating the direct and indirect influence of satisfaction with the micro learning environment on professional identity is vital.

**Figure 1:**
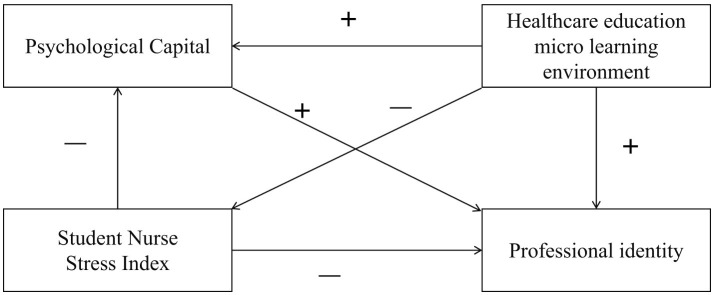
Hypothetical model.

Previous studies have investigated various factors such as PsyCap, stress as measured on the Student Nurse Stress Index, satisfaction with the micro learning environment, and professional identity. However, these studies often examine these factors in isolation or focus solely on limited correlations without exploring their interrelationships, particularly within the context of nursing interns. A more comprehensive approach is needed to explore how these variables interact within a cohesive framework. To address this gap, this study adopts SCCT as its theoretical foundation. SCCT suggests that career development is influenced by the interaction of cognitive variables, personal characteristics, and environmental factors. According to SCCT, professional identity is a key developmental outcome resulting from the interaction of personal characteristics, environmental factors, and individual behaviors. Specifically, PsyCap, as a personal characteristic, aids in stress management, while stress, as an environmental factor, interacts with PsyCap, influencing professional identity formation. Additionally, the learning environment, another external factor, plays a vital role in shaping professional identity. Guided by SCCT, this study aims to explore the interrelationships among PsyCap, stress, the learning environment, and professional identity in nursing interns, using structural equation modeling (SEM) to examine how these factors interact and contribute to the development of professional identity. Based on this theory, the following hypotheses were tested (Figure 1).

**Hypothesis 1:** PsyCap exerts a notable direct impact on professional identity.
**Hypothesis 2:** The student nurse stress index shows that stress has a considerable direct negative and overall influence on professional identity.
**Hypothesis 3:** Satisfaction with the healthcare education micro learning environment significantly positively contributes to professional identity.
**Hypothesis 4:** PsyCap mediates between student nurse stress levels, satisfaction with the healthcare education micro learning environment and professional identity.

With these hypotheses, this study aims to explore how PsyCap, stress, and the micro-learning environment influence the professional identity of nursing interns. It will contribute to the literature by clarifying the relationships between these factors and offering valuable insights for both hospital managers and nursing schools. The findings will highlight strategies to enhance PsyCap, manage stress, and improve the learning environment, all of which are crucial for fostering professional identity and bridging the gap between nursing education and clinical practice.

## 2 Methods

### 2.1 Study design

To investigate, a cross-sectional study was devised to explore the professional identity of nursing students doing internships and to test the hypotheses for estimating the effects of PsyCap, and stress between micro learning environment and professional identity. It was conducted according to the guideline for Strengthening the Report of Observational Studies in Epidemiology.

### 2.2 Participants

Participants were 388 nursing interns recruited from 8 comprehensive teaching hospitals using convenience sampling from March to April 2024 across five cities in Hubei Province of China. The study enrolled participants who met the following inclusion criteria: (a) nursing interns; (b) Education: full-time nursing college and above; (c) age ≥ 20 years old; (d) agreed to participate in the study.

The sample size was determined using Kyriazos' recommendations for correlational studies of 5–10 times the number of variables plus 20% for invalid questionnaires (Kyriazos, 2018). As our study had 21 variables this gave a minimum of 252 participants.

### 2.3 Variables

#### 2.3.1 Descriptive information questionnaire

A 10 item questionnaire was designed based on the literature review covering demographic information such as age, gender, family size, education level and related professional experience and identity such as workplace violence history, occupational injury history, clinical safety, voluntary choice of nursing career, medical family background, and nursing role model.

#### 2.3.2 PsyCap

PsyCap among nursing interns was assessed using the Chinese version of the Psychological Capital Questionnaire (C-PCQ) (Hong and Hao, 2010; Luthans et al., 2007), which contains 20 items distributed across four subscales: self-efficacy, hope, resilience, and optimism. Example items included “I feel confident helping to set targets/goals in my work area”, “Right now I see myself as being pretty successful at work”, “I usually take stressful things at work in stride”, and “I always look on the bright side of things regarding my job”. Respondents rated each item on a 6-point Likert scale, ranging from 1 (strongly disagree) to 6 (strongly agree). Higher scores indicate greater PsyCap. The Cronbach's alpha coefficient for this study was calculated at 0.971.

#### 2.3.3 Student nurse stress index scale

Stress levels were assessed using the Chinese version of the Student Nurse Stress Index scale (SNSI-CHI) (Jones and Johnston, 1999). This scale comprises 22 items categorized into four dimensions: academic load, clinical concerns, interface worries, and personal problems. Example items included “Difficulty of classwork”, “Client attitudes (to student)”, “Peer competition”, “Physical health (family)”. Responses are measured on a 5-point Likert scale, ranging from 1 (minimal stress) to 5 (highly stressful). Total scores range from 22 to 110, with higher scores indicating greater stress levels. The SNSI-CHI has demonstrated strong reliability and validity in Chinese contexts (Guo et al., 2018), as confirmed by a Cronbach's alpha coefficient of 0.963 in this study.

#### 2.3.4 Healthcare education micro learning environment measure

Satisfaction with the learning environment was measured using the Healthcare Education Micro Learning Environment Measure (HEMLEM) (Isba et al., 2020; Zhang et al., 2022) which consists of 12 items covering two dimensions: staff attitudes and behaviors, and teaching quality. Example items included “My input was valued on this placement”, “I was able to meet my learning objectives on this placement”. The HEMLEM is scored using a 5-point Likert scale, the overall score thus ranged between 12 and 60. Participants who achieved a total score exceeding 36 were deemed to be satisfied. The Cronbach's alpha coefficient in this study was 0.959.

#### 2.3.5 Professional identity

The Chinese version of Professional Identity Scale (Tyler and McCallum, 1998; Cai, 2004) was utilized to evaluate the professional identity of nursing interns. Example items include, “My present job makes me feel very proud”. The scale consists of 10 items assessed on a 5-point Likert scale ranging from 1 (completely incongruous) to 5 (completely congruous). Professional identity levels were categorized as disapproval for scores between 10 and 20, general identity for scores between 21 and 40, and high identity for scores between 41 and 50. The Cronbach's alpha for this study was 0.941.

### 2.4 Data collection

Data was collected using an online survey platform called Wenjuanxing. Firstly, The survey questionnaire was crafted on the Wenjuanxing platform, and a QR code was subsequently generated for quick access. Secondly, the researchers informed the nursing director of each hospital about the survey purpose and obtained their permission to recruit nursing interns, the QR code was sent to directors who agreed to participate. Thirdly, the nursing director could either distribute the QR code with informed consent via the social media platform (WeChat). Nursing interns could scan the QR code and complete the questionnaires on their mobile phones if they consented to engage in the study. It took approximately 5 to 10 min to finish the electronic questionnaire. After submission of the questionnaires, researchers could access and download the data from the online platform for subsequent analysis. The invalid criteria included: (a) submissions made within 90 s; (b) absence of fundamental values; and (c) identification of outliers.

### 2.5 Ethical consideration

Ethical approval for the study was obtained from the Taihe Hospital ethics committee (review number 2024KS14). No personally identifiable information was gathered and participants were granted the autonomy to discontinue their participation in the study at any time.

### 2.6 Statistical analysis

Data analysis was conducted using IBM SPSS 26.0 on Windows. Participant demographic characteristics were presented as frequency, percentage, mean, and standard deviation. Group differences were evaluated using independent sample *t*-tests and ANOVA. The relationship between variables was examined using Pearson correlation coefficients. A *p*-value < 0.05 was considered statistically significant. Stepwise multiple linear regression was applied to identify factors influencing nursing interns' professional identity.

Structural equation modeling (SEM) was utilized in this study using AMOS 24.0. Various fit indices, including the chi-square/degrees of freedom ratio (χ2/*df*), standardized root mean square residual (SRMR), root mean square error of approximation index (RMSEA), goodness-of-fit index (GFI), adjusted goodness-of-fit index (AGFI), normed fit index (NFI), comparative fit index (CFI), and Tucker–Lewis Index (TLI). A χ2/*df* value of below 3.00 indicates a good model fit. Similarly, SRMR and RMSEA values below 0.05 suggest a satisfactory model fit. Additionally, GFI, AGFI, NFI, CFI, and TLI values exceeding 0.90 indicate a good model fit. Maximum likelihood estimation method for covariance matrices was applied, and Bootstrapping (5,000 times) was used to test the indirect and total effects of the model.

## 3 Results

### 3.1 Sociodemographic characteristics

A total of 421 questionnaires were collected, with 23 were excluded for submission within 2 min and 10 were removed as outliers, resulting in an effective response rate of 92.16%. The final sample included 388 nursing interns, with an average age of 21.28 ± 1.02 years. Among the participants, 85.1% (*n* = 330) were female, and 67.5% (*n* = 262) were the only child in their family. The nursing education levels were 43.0% (*n* = 167) undergraduate, 11.6% (*n* = 45) associate-to-undergraduate, and 45.4% (*n* = 176) college. Nursing interns who are only children, have a history of workplace violence or occupational injury, and perceive the hospital nursing environment as safe tend to have a lower level of professional identity. In contrast, nursing interns who voluntarily choose a nursing career and have a nursing role model tend to have a higher level of professional identity. Nursing interns' professional identity score and their complete Sociodemographic characteristics are presented in Table 1.

**Table 1:** Participants' characteristics (*N* = 388).

**Demographics**	***N* (%)**	**Professional identity**		
		**Mean (Mean** ±**SD)**	***t/F***	***P***
**Age (years), Mean** **±SD**	21.28 ± 1.02			
**Gender**				
Male	58 (14.9)	3.63 ± 0.80	0.82	0.414
Female	330 (85.1)	3.54 ± 0.74		
**Only child in family**				
Yes	126 (32.5)	3.44 ± 0.81	−2.17	0.031
No	262 (67.5)	3.61 ± 0.71		
**Nursing education level**				
Full-time undergraduate program	167 (43)	3.48 ± 0.75	1.77	0.172
Full-time undergraduate program for associate degree holders	45(11.6)	3.59 ± 0.73		
Full-time college	176 (45.4)	3.63 ± 0.75		
**Workplace violence history**				
Yes	19 (4.9)	2.95 ± 0.71	−3.71	0.000
No	369 (95.1)	3.59 ± 0.74		
**Occupational injury history**				
Yes	113 (29.1)	3.32 ± 0.75	−4.00	0.000
No	275 (70.9)	3.65 ± 0.73		
**Is hospital nursing environment safe**				
Safe	121 (31.2)	3.99 ± 0.73	27.83	0.000
Relatively safe	215 (55.4)	3.43 ± 0.63		
Relatively unsafe	35 (9.0)	3.05 ± 0.69		
Unsafe	17 (4.4)	3.14 ± 0.91		
**Voluntary choice of nursing career**				
Yes	292(75.3)	3.71 ± 0.69	7.60	0.000
No	96 (24.7)	3.09 ± 0.72		
**Medical family background**				
Yes	115 (29.6)	3.55 ± 0.74	−0.10	0.923
No	273 (70.4)	3.56 ± 0.75		
**Nursing role model**				
Yes	215 (55.4)	3.77 ± 0.66	6.46	0.000
No	173 (44.6)	3.30 ± 0.77		

### 3.2 Confirmatory factor analysis of measured variables

The assessment of convergent validity, based on average variance extracted (AVE) and composite reliability (CR), yielded satisfactory results (see Supplementary Table 1). Consequently, the factor loadings for the four factors of PsyCap, self-efficacy, hope, and resilience ranged from 0.78 to 0.86, 0.70 to 0.913, 0.80 to 0.86, and 0.80 to 0.86, respectively. For the four dimensions of SNSI-CHI, the factor loadings were in the range of 0.68–0.85. The two dimensions of HEMLEM exhibited factor loadings range from 0.73 to 0.88. Additionally, the factor loadings for professional identity fell within the range of 0.67–0.86. Importantly, all factor loadings surpassed 0.60, indicating strong measurement reliability. The AVE values of the latent variables ranged from 0.56 to 0.82, while the composite reliability (CR) values varied between 0.87 and 0.94. These results confirm the presence of internal consistency reliability and convergent validity.

### 3.3 Common-method bias

All data from C-PCQ, SNSI-CHI, HEMLEM, and the Professional Identity Scale were tested for common method bias using Harman's one factor-test. The unrotated exploratory factor analysis extracted eight factors with characteristic roots >1. The maximum factor variance explained was 24.80% (< 40%). Thus, there was no common-method severe bias in this study.

### 3.4 PsyCap, stress, micro learning environment, professional identity, and their associations

Table 2 presents the correlation among the study variables, their components and descriptive statistics. The mean scores for PsyCap, stress, micro learning environment, and professional identity were 91.10 ± 15.90, 60.44 ± 17.41, 47.58 ± 7.20, and 35.57 ± 7.47, respectively. Correlation analyses revealed a negative association between professional identity and stress (*r* = −0.42, *P* < 0.001). Whereas, it demonstrated a positive correlation with PsyCap (*r* = 0.49, *P* < 0.001) and micro learning environment and their dimensions (*r* = 0.65, *P* < 0.001).

**Table 2:** Means, standard deviations, and correlations among the study variables (*N* = 388).

**Variables**	**Mean ±SD**	**1**	**2**	**3**	**4**	**5**	**6**	**7**	**8**	**9**	**10**	**11**	**12**	**13**	**14**
1. PsyCap	91.10 ± 15.90	1													
2. Self-efficacy	4.62 ± 0.84	0.909^**^	1												
3. Hope	4.56 ± 0.83	0.950^**^	0.801^**^	1											
4. Resilience	4.50 ± 0.85	0.938^**^	0.783^**^	0.872^**^	1										
5. Optimism	4.51 ± 0.98	0.873^**^	0.704^**^	0.797^**^	0.797^**^	1									
6. Student Nurse Stress Index Scale	60.44 ± 17.41	−0.414^**^	−0.338^**^	−0.399^**^	−0.403^**^	−0.398^**^	1								
7. Academic load	2.94 ± 0.86	−0.382^**^	−0.304^**^	−0.372^**^	−0.368^**^	−0.377^**^	0.894^**^	1							
8. Clinical concerns	2.55 ± 0.87	−0.394^**^	−0.334^**^	−0.386^**^	−0.389^**^	−0.337^**^	0.913^**^	0.727^**^	1						
9. Interface worries	3.00 ± 0.86	−0.377^**^	−0.288^**^	−0.356^**^	−0.374^**^	−0.395^**^	0.933^**^	0.825^**^	0.784^**^	1					
10. Personal problems	2.37 ± 0.95	−0.327^**^	−0.289^**^	−0.311^**^	−0.301^**^	−0.306^**^	0.835^**^	0.654^**^	0.738^**^	0.676^**^	1				
11. Healthcare Education Micro Learning Environment Measure	47.58 ± 7.20	0.484^**^	0.414^**^	0.494^**^	0.422^**^	0.454^**^	−0.430^**^	−0.373^**^	−0.429^**^	−0365^**^	−0.378^**^	1			
12. Staff attitudes and behaviors	3.95 ± 0.66	0.446^**^	0.382^**^	0.455^**^	0.386^**^	0.424^**^	−0.411^**^	−0.347^**^	−0418^**^	−0.350^**^	−0.361^**^	0.959^**^	1		
13. Teaching quality	3.98 ± 0.60	0.479^**^	0.410^**^	0.490^**^	0.421^**^	0.443^**^	−0.409^**^	−0.365^**^	−0.401^**^	−0.346^**^	−0.359^**^	0.950^**^	0.822^**^	1	
14. Professional identity	35.57 ± 7.47	0.487^**^	0.360^**^	0.490^**^	0.443^**^	0.539^**^	−0.423^**^	−0.413^**^	−0.338^**^	−0.451^**^	−0.289^**^	0.652^**^	0.606^**^	0.640^**^	1

^**^P < 0.01.

SD, standard deviation.

### 3.5 Determinants of professional identity among nursing interns

To identify factors influencing professional identity among nursing interns, all variables were included and analyzed using multiple linear regression. The findings indicated the absence of multicollinearity among the independent variables based on collinearity diagnosis. The results showed that seven factors significantly influence professional identity, including not voluntary choice in nursing, no nursing role model, relatively safe working environment, relatively unsafe working environment, PsyCap, stress levels and micro learning environment (Table 3). These factors accounted for 53.1% of the variability observed in professional identity scores (*R*^2^ = 0.531, *P* < 0.001).

**Table 3:** Multiple linear regression analysis for the factors of professional identity scores (*N* = 388).

**Independent variables**	**Model 1**				**Model 2**			
	β	**95%CI**		***P***	β	**95%CI**		***P***
		**Lower**	**Upper**			**Lower**	**Upper**	
No workplace violence history	0.321	0.003	0.639	0.048	0.165	−0.099	0.429	0.219
Not voluntary choice in nursing	−0.380	−0.538	−0.222	< 0.001	−0.24	−0.337	−0.071	0.003
No nursing role model	−0.295	−0.428	−0.162	< 0.001	−0.143	−0.255	−0.031	0.012
Full-time college	0.167	0.010	0.323	0.037	0.106	−0.024	0.236	0.108
Relatively Safe working environment	−0.469	−0.615	−0.322	< 0.001	−0.152	−0.282	−0.021	0.023
Relatively unsafe working environment	−0.685	−0.945	−0.426	< 0.001	−0.319	−0.541	−0.097	0.005
Unsafe working environment	−0.585	−0.913	−0.257	0.001	0.259	−0.537	0.019	0.068
PsyCap					0.185	0.104	0.266	< 0.001
Student nurse stress index scale					−0.092	−0.170	−0.013	0.022
Health education micro learning environment measurement					0.477	0.364	0.589	< 0.001
Adjusted R^2^	0.310				0.531			
*F*	13.840				27.370			
*p*	< 0.001				< 0.001			

β, unstandardized coefficient; CI, confidence interval.

### 3.6 Structural equation modeling results

**Figure 2:**
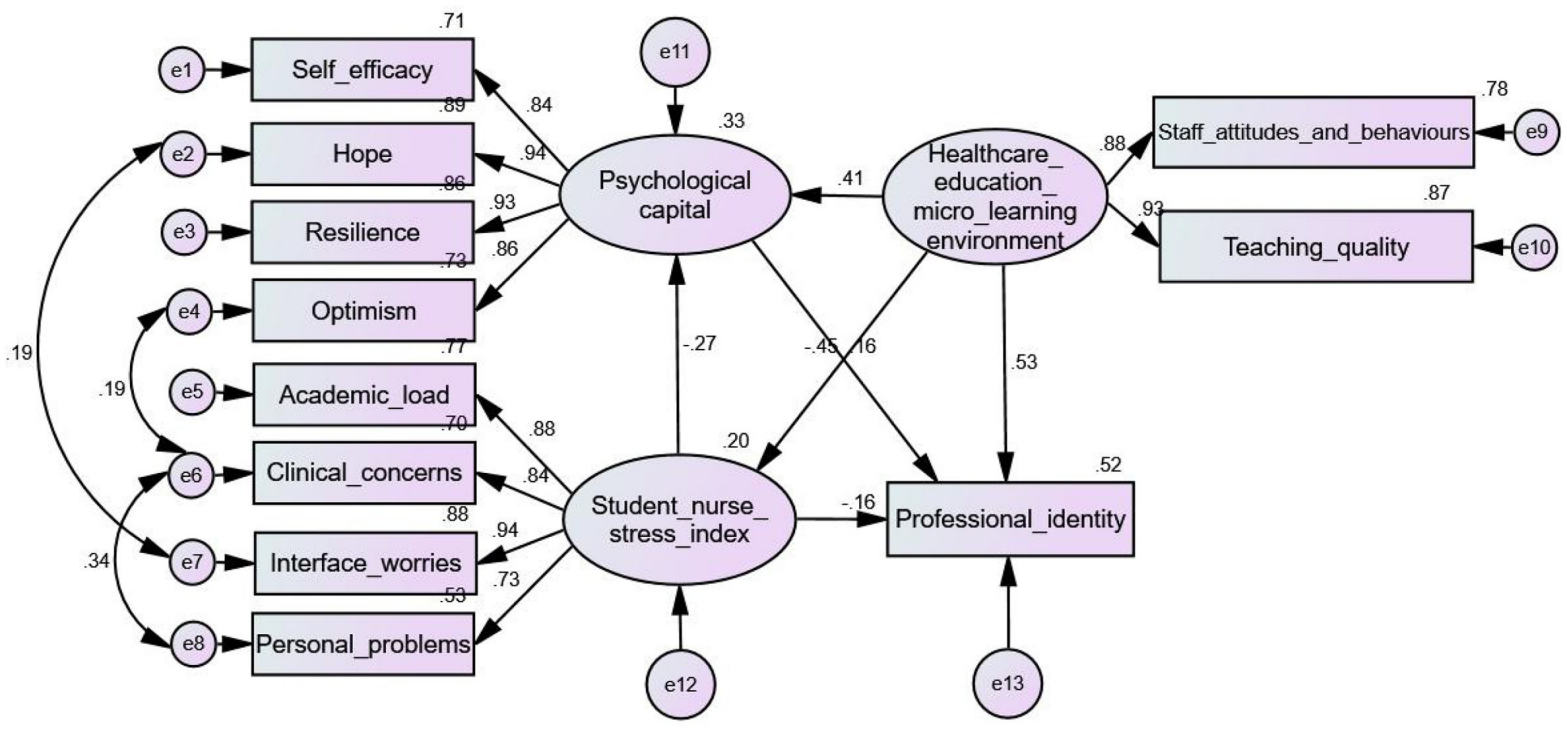
Standardized parameter estimates for the final structure model.

After conducting the regression analysis, a structural equation model was employed to explore the effects of PsyCap, stress, micro-learning environment, and professional identity on nursing interns (Figure 2). The fitting index of the mediation model was good (χ^2^/*df* = 2.933, SRMR = 0.032, GFI = 0.953, AGFI = 0.913, NFI = 0.971, TLI = 0.970, CFI = 0.980, RMSEA = 0.071), indicating that the model was acceptable, as shown in Table 4. PsyCap directly affects professional identity (β = 0.16, *P* < 0.01), which validates Hypothesis 1. Student nurse stress index scores have a negative effect on professional identity (β = −0.20, *P* < 0.01), which supports Hypothesis 2. The Healthcare education micro learning environment affects professional identity (β = 0.69, *P* < 0.001), supporting Hypothesis 3.

**Table 4:** Analyzing the model fit for the modified model to the hypothetical model.

**Model**	***χ2*(*P*)**	***df***	***χ^2^/df***	**SRMR**	**GFI**	**AGFI**	**NFI**	**TLI**	**CFI**	**RMSEA**
Reference	>0.05		1~3	< 0.05	0.9–1	0.9–1	0.9–1	0.9–1	0.9–1	< 0.08
Fitted Model	105.576(0.00)	36	2.933	0.032	0.953	0.913	0.971	0.970	0.980	0.071

*df* , degree of freedom; SRMR, standardized root mean square residual; GFI, goodness-of-ft index; AGFI, adjusted goodness-of-fit index; NFI, normed fit index; TLI, Tucker–Lewis Index; CFI, comparative fit index; RMSEA, root mean square error of approximation index.

Moreover, the 95% confidence intervals (CIs) in the mediation model did not include zero, indicating that all indirect effects were significant. These findings support Hypothesis 4 by demonstrating that the microlearning environment in healthcare education indirectly influences professional identity through PsyCap (β = 0.09, *P* < 0.01), the student nurse stress index score (β = 0.09, *P* < 0.01), and their combined effect (β = 0.03, *P* < 0.01), collectively accounting for 22.6% of the total effect of the microlearning environment on professional identity. Furthermore, the student nurse stress index score exerted an indirect effect on professional identity via PsyCap (β = −0.04, *P* < 0.01), also explaining 22.6% of the total effect of the student nurse stress index score on professional identity. These results provide additional confirmation for Hypothesis 4. The model outlined in the study explained 52.1% of the variation in professional identity. Table 5 provides a detailed breakdown of the model's overall, direct, and mediated impacts.

**Table 5:** Total, direct, and indirect effects of each path in this model.

**Estimate**	**β**	**SE**	**BC 95% CI**		***P***
			**Lower**	**Upper**	
**Total effects**					
HEMLEM → SNSIS	−0.446	0.047	−0.534	−0.351	0.000
HEMLEM → PC	0.527	0.060	0.379	0.631	0.001
HEMLEM → PI	0.687	0.040	0.603	0.757	0.000
SNSIS → PC	−0.266	0.059	−0.378	−0.143	0.001
SNSIS → PI	−0.200	0.054	−0.305	−0.094	0.000
PC → PI	0.161	0.063	0.050	0.296	0.004
**Standardized direct effects**					
HEMLEM → SNSIS	−0.446	0.047	−0.534	−0.351	0.000
HEMLEM → PC	0.408	0.057	0.288	0.513	0.000
HEMLEM → PI	0.532	0.064	0.402	0.649	0.000
SNSIS → PC	−0.266	0.059	−0.378	−0.143	0.001
SNSIS → PI	−0.157	0.051	−0.257	−0.058	0.002
PC → PI	0.161	0.063	0.050	0.296	0.004
**Standardized indirect effects**					
Total HEMLEM → PI	0.155	0.040	0.083	0.238	0.000
HEMLEM → PC → PI	0.085	0.039	0.027	0.180	0.003
HEMLEM → SNSIS → PI	0.090	0.031	0.036	0.161	0.002
HEMLEM → SNSIS → PC → PI	0.025	0.013	0.006	0.057	0.003
SNSIS → PC → PI	−0.043	0.023	−0.100	−0.011	0.004
HEMLEM → SNSIS → PC	0.119	0.028	0.065	0.176	0.000

PC, PsyCap; SNSIS, Student Nurse Stress Index; HEMLEM, Healthcare Education Micro Learning Environment Measure; PI, Professional Identity.

## 4 Discussion

The results of this study reinforce the theoretical understanding of SCCT and show that PsyCap, stress, and the micro learning environment are significantly strongly correlated with professional identity among nursing interns. In the multivariate analyses, not voluntary choosing nursing as a career, not having a nursing role model, perceived safety of the working environment, PsyCap, stress measured on the student nurse stress index, and micro learning environment were the main factors associated with professional identity. Structural modeling results showed that PsyCap mediated the association between the healthcare education micro learning environment, student nurse stress, and professional identity. All hypotheses regarding the relationships between variables in this study were validated.

In this study, nursing interns' professional identity score was at a medium level, consistent with previous studies (Luo and Mao, 2022). Meanwhile, compared to novice nurses, the scores of nursing interns were slightly lower (Zhong et al., 2024), which may be because nursing interns lack specific clinical duties, primarily observing and practicing clinical skills under mentor supervision. Novice nurses, however, are more actively engaged in clinical tasks and receive advanced training, which can improve their professional identity (Lin et al., 2024). Contrary to our results, Gilvari discovered a strong professional identity among Iranian nursing students which may be due to the different conditions pertaining in different countries and regions (Gilvari et al., 2022). Further studies should be conducted to thoroughly investigate professional identity across various settings.

Based on the regression results, four demographic factors can influence the professional identity among nursing interns. Nursing interns who did not choose nursing as a career exhibited lower professional identity than their counterparts for whom nursing was their career choice which is consistent with former studies (Zeng et al., 2022). For nursing interns who voluntarily choose nursing, their career interests and aspirations align with the profession, fostering increased interest and motivation in their studies. In the early stages of vocational education, nurse educators should focus on students who did not voluntarily choose nursing, providing them with a thorough understanding of the profession, setting role models, and arranging clinical rotations across hospitals of various levels (Mei et al., 2022; Zeng et al., 2022). Similarly, nursing interns who lack nursing role models exhibit lower levels of professional identity than those with nursing role models. Good role models, recognized as critical factors in developing students' professional identity, can inspire nursing students to reconsider their preconceptions about the nursing profession, expand their perception of its possibilities, and consequently bolster their professional identity (Baldwin et al., 2017; Felstead and Springett, 2016). Both clinical supervisors and academic faculty serve as crucial role models for nursing interns (Fitzgerald, 2016). Clinical supervisors should demonstrate responsibility and kindness toward nursing interns, trusting their capabilities, and displaying empathy and compassion toward patients (Vabo et al., 2022). Similarly, academic teachers should be proficient in theoretical and practical teachings, actively engaging students in the class to foster a supportive learning environment (Vabo et al., 2022). This approach aids in shaping a professional image, bolstering learning motivation, promoting focus, and minimizing insecurity (Felstead and Springett, 2016). Nursing schools can boost professional identity by arranging museum visits, teaching nursing history, and exposing students to positive nursing media, facilitating historical and societal role models for nursing students (Kelly et al., 2017; Li et al., 2022). Both relatively safe and unsafe environments can negatively impact nursing interns' professional identity. Among the surveyed students, 4.9% reported a history of workplace violence, while 29.7% reported experiencing occupational injuries, contributing to decreased perceptions of workplace safety. Those risk factors in the working environments can decrease nursing interns' professional identity (Lin et al., 2024; Yang et al., 2023). Efforts should be made to prevent workplace violence and occupational injuries in healthcare settings. Adopting a “safe hospital” policy and offering education and support to nursing interns in such situations are imperative steps to take (Yang et al., 2023). In conclusion, nursing schools should collaborate with hospital staff to improve nursing students' professional identity by supporting those who did not select nursing voluntarily, establishing role models for students, and ensuring a safe working environment for nursing interns.

Our results support the hypotheses suggesting that PsyCap significantly and directly influences the professional identity of nursing interns, aligning with SCCT, which highlights the importance of personal characteristics in career development. Additionally, PsyCap serves as a mediator between stress and professional identity, reinforcing SCCT's concept that personal characteristics (like PsyCap) moderate the effects of environmental stressors on career-related outcomes. The results align with a prior investigation centered on medical interns (Liu et al., 2023). The PsyCap of surveyed nursing interns was medium to high, consistent with prior studies on nurses (Yao et al., 2023). Nursing interns with elevated PsyCap demonstrate heightened levels of professional identity. PsyCap, characterized by a positive psychological state, plays a vital role in shaping individuals' coping strategies and overall psychological wellbeing during periods of environmental changes and events (Luthans et al., 2007). When facing complex work environments and challenges, nursing interns with high PsyCap exhibit heightened positive emotions, enhanced coping abilities, and intrinsic motivation (Belle et al., 2022), fostering a firm professional identity. The PsyCap can mediate between stress and professional identity. Each component of PsyCap may influence stress differently: efficacy can moderate stress's effects, optimism positively impacts nurses' quality of life, hope predicts stress levels and resilience is crucial for maintaining mental health (Jarvis-Isaac, 2023). Nursing interns with high PsyCap are optimistic about the nursing discipline development. This, facilitates continuous learning and achievement in clinical settings while alleviating academic and life pressures, reducing stress levels, and enhancing professional identity attainment (Javaheri, 2017). Another critical point is PsyCap has been considered malleable, indicating that it can be cultivated in individuals over time (Javaheri, 2017). This may be achieved through targeted training or group interventions (Flinkman et al., 2023; Luthans et al., 2008). Professional career counselors can work with nursing students to develop positive PsyCap helping them to decrease stress and improve their professional identity.

The student nurse stress index scores in this study were moderate. Moreover, The results highlight stress as a predictor of professional identity among nursing interns, with SCCT suggesting that stress, as an environmental factor, shapes career outcomes, including professional identity. These findings are consistent with earlier studies (Sun et al., 2016; Zhao et al., 2021), indicating a negative correlation between high stress levels and professional identity. Stress can positively impact academic performance, fostering active learning and professional growth, but also cause negative physical, psychological, and social symptoms that undermine performance and negatively affect professional identity construction (Araújo et al., 2023). In this study, the interface worries dimension scores highest, followed by the academic load dimension, suggesting that nursing interns' stress mainly stems from interface worries and academic loads. The Interface worries dimension measures stress related to peer competition, professional attitudes, school responsiveness, lack of recognition, and limited personal time (Guo et al., 2018). The academic workload dimension measures course burden, learning difficulties, exam results, fear of academic failure, and clarity of personal goals. One approach is to encourage universities to prioritize creating a supportive learning environment, offering timely feedback and recognition, providing guidance for learning challenges, integrating career planning into the curriculum, promoting work-life balance, and clarifying academic goals to reduce fear of failure.

Furthermore, this model also revealed that a good healthcare education micro learning environment positively affected nursing interns' professional identity, as SCCT emphasizes that the environment shapes career development by providing learning experiences that influence self-efficacy and career-related behaviors. Previous studies have reported that the clinical internship environment can influence professional identity (Zeng et al., 2022). The learning environment comprises diverse micro learning environments, encompassing people, physical space, resources, opportunities, and emotional and social elements (Hasnain et al., 2024). This study is the first research to investigate the effect of a micro learning environment on professional identity among nursing interns. Staff attitudes and behavior can foster a positive work environment and a constructive culture, enabling nurses to serve as role models for nursing interns by delivering high-quality patient care, a critical factor in shaping professional identity (Fitzgerald and Clukey, 2021). High teaching quality enables nursing interns to be assigned reasonable internship tasks, allowing them to apply their learned knowledge and skills during internships, thereby achieving learning objectives, which benefits the cultivation of their professional identity (Wu et al., 2020). Moreover, PsyCap mediates between the healthcare education micro-learning environment and professional identity, aligning with prior studies (Wu et al., 2020). On the one hand, A positive clinical learning environment can enhance nursing interns' resilience, a crucial aspect of PsyCap that fosters professional identity formation (Xu et al., 2024). On the other hand, PsyCap serves as a stable positive personal resource (Javaheri, 2017); it was found that elevated PsyCap boosts personal confidence and motivation to pursue success, enhancing the ability to effectively cope with challenging situations, resulting in a higher level of professional identity (Luthans et al., 2007; Qiu et al., 2019; Zhao et al., 2021). In general, the healthcare education micro-learning environment has a significant impact on nursing interns' professional identity. To enhance their professional identity, efforts should be made to establish a conducive clinical learning environment for them. Teaching hospitals should cultivate a supportive work environment by fostering a caring atmosphere, offering them assistance and encouragement, and alleviating their workload (Tao et al., 2023). Additionally, improving teaching quality involves rigorous selection, assessment, standardized training for clinical mentors, and tailoring internship schedules for nursing interns. Moreover, nursing schools should follow students during internships and provide support for them.

## 5 Limitations and future research

We recognize several limitations in this study. The study's cross-sectional design poses challenges in establishing direct causal relationships between variables. Another limitation is the exclusive reliance on self-reported questionnaire surveys for data collection, which may introduce biases such as social desirability or recall errors. Furthermore, using convenience sampling to recruit nursing interns from various hospitals in the Hubei Province of China might offer a partial rather than comprehensive representation of all nursing students thus limiting generalization. Although the study concentrated on nursing interns' professional identity utilizing the Social Cognitive Career Theory and demographic variables, the phenomenon complexity suggests that other potentially influential factors in professional identity formation might have yet to be considered.

Future studies could employ longitudinal and qualitative methods to gain deeper insights into the causal relationships between the variables examined in this research. Engaging diverse stakeholders, such as nursing interns, academic instructors, and clinical mentors, through qualitative approaches would help explore the underlying mechanisms in greater detail. Additionally, leveraging longitudinal data could provide a more comprehensive understanding of how PsyCap, stress, the microlearning environment in healthcare education, and professional identity interact over time. Further research and interventions on PsyCap, stress, and the healthcare education micro-learning environment could provide insights into their impact on professional identity, offering empirical evidence to support the enhancement of nursing interns' professional identity and contribute to the stability of the nursing workforce.

## 6 Conclusion

This study contributes to current knowledge by conducting structural analysis to explore the relationship between PsyCap, stress, healthcare education micro-learning environment, and professional identity. Collaborative efforts between nursing schools and clinical departments are recommended to provide role models and implement training interventions to enhance nursing students' PsyCap and stress management skills. Creating a satisfied and safe working environment is crucial for fostering professional identity among nursing interns. Future research should focus on longitudinal studies to further understand the mediating mechanisms of PsyCap in the relationship between student nurse stress, healthcare education micro-learning environment, and professional identity.

## Data Availability

The raw data supporting the conclusions of this article will be made available by the authors, without undue reservation.

## References

[B1] American Psychological Association (2018). Stress. Washington, DC: American Psychological Association.

[B2] Araújo A. A. C. Godoy S. D. Maia N. M. F. E. Oliveira R. M. D. Vedana K. G. G. Sousa Á. D. F. L.. (2023). Positive and negative aspects of psychological stress in clinical education in nursing: a scoping review. Nurse Educ. Today 126:105821. 10.1016/j.nedt.2023.105821 37080012

[B3] Baldwin A. Mills J. Birks M. Budden L. (2017). Reconciling professional identity: a grounded theory of nurse academics' role modelling for undergraduate students. Nurse Educ. Today 59, 1–5. 10.1016/j.nedt.2017.08.010 28898727

[B4] Belle M. A. Antwi C. O. Ntim S. Y. Affum-Osei E. Ren J. (2022). Am I gonna get a job? Graduating students' psychological capital, coping styles, and employment anxiety. J. Career Dev. 49, 1122–1136. 10.1177/08948453211020124

[B5] Cai C. J. (2004). “Discussion on the role of fuzziness and professional identity in preschool teachers' professional roles,” *Proceedings of the Academic Research and Creation Achievements of the 92nd Annual Academic Year of the School of Humanities and Social Sciences* (Chaoyang, Beijing: Chaoyang University of Science and Technology), 355–380.

[B6] Chang T. Jiang X. Wei J. Zhao J. Li Z. Li H. (2023). Mediating effects of psychological capital on the relationship between workplace violence and professional identity among nurses working in Chinese public psychiatric hospitals: a cross-sectional study. BMJ Open 13:e65037. 10.1136/bmjopen-2022-065037 36599638 PMC9815003

[B7] Ding X. Yang L. Huang Y. Yang H. Zhao X. Jiang Q.. (2024). The relationship between perceived social support and bullying behavior in nursing education among nursing students: the mediating role of positive psychological capital. Perspect. Psychiatric Care. 2024:6642262. 10.1155/2024/6642262

[B8] Elliott R. Fry M. (2021). Psychological capital, well-being, and patient safety attitudes of nurses and midwives: a cross-sectional survey. Nurs. Health. Sci. 23, 237–244. 10.1111/nhs.12808 33382147

[B9] Felstead I. S. Springett K. (2016). An exploration of role model influence on adult nursing students' professional development: a phenomenological research study. Nurse Educ. Today 37, 66–70. 10.1016/j.nedt.2015.11.014 26673614

[B10] Fitzgerald A. (2020). Professional identity: a concept analysis. Nurs. Forum 55, 447–472. 10.1111/nuf.12450 32249453

[B11] Fitzgerald A. Clukey L. (2021). Professional identity in graduating nursing students. J. Nurs. Educ. 60, 74–80. 10.3928/01484834-20210120-04 33528577

[B12] Fitzgerald A. M. (2016). The Experience of Professional Identity Development in Graduating Nursing Students. Greeley, CL: University of Northern Colorado.

[B13] Flinkman M. Coco K. Rudman A. Leino-Kilpi H. (2023). Registered nurses' psychological capital: a scoping review. Int. J. Nurs. Pract. 29:e13183. 10.1111/ijn.13183 37485748

[B14] Giddens J. F. (2021). Concepts of Nursing Practice. Canada: Elsevier Publishing.

[B15] Gilvari T. Babamohamadi H. Paknazar F. (2022). Perceived professional identity and related factors in Iranian nursing students: a cross-sectional study. BMC Nurs. 21, 279–287. 10.1186/s12912-022-01050-6 36229807 PMC9559545

[B16] Guo L. Yu S. Zhu Y. Guo Y. Li L. Ding X.. (2018). Reliability and validity of the Chinese version of the student nurse stress index scale (SNSI-CHI). Chin. J. Behav. Med. Brain Sci. 27, 937–941. 10.3760/cma.j.issn.1674-6554.2018.10.01530704229

[B17] Hasnain M. Iqbal M. Z. Iqbal N. Khan A. H. Hameed S. (2024). Microlearning environment of orthodontic postgraduate training programmes in Pakistan: a multicentre cross-sectional study. J. Coll. Physicians Surg. Pak. 34, 91–96. 10.29271/jcpsp.2024.01.91 38185968

[B18] Hong L. Hao Z. (2010). The relationships among psychological capital, job burnout and turnover intention in 466 nurses. Chin. J. Nurs. 45, 933–935. 10.3761/j.issn.0254-1769.2010.10.027

[B19] Huang Z. Qiu X. Yan J. Liao D. Huang H. Fu Y.. (2024). Structural equation modeling for associated factors with patient safety behaviors among nursing interns: a cross-sectional study based on the capability opportunity motivation-behavior model. Nurse Educ. Today 132:105992. 10.1016/j.nedt.2023.105992 37890194

[B20] Isba R. Rousseva C. Woolf K. Byrne-Davis L. (2020). Development of a brief learning environment measure for use in healthcare professions education: the Healthcare Education Micro Learning Environment Measure (HEMLEM). BMC Med. Educ. 20:110. 10.1186/s12909-020-01996-8 32272934 PMC7146917

[B21] Jarvis-Isaac R. J. (2023). Exploring Relationships between Psychological Capital and Perceived Stress among Newly Graduated Nurses. Columbia: Walden University.

[B22] Javaheri A. (2017). Psychological Capital: An Internal Resource for Counseling Students Coping with Academic and Clinical Stress. Williamsburg, VA: College of William & Mary.

[B23] Jones M. C. Johnston D. W. (1999). The derivation of a brief student nurse stress index. Work Stress 13, 162–181. 10.1080/026783799296129

[B24] Kelly J. Watson R. Watson J. Needham M. Driscoll L. O. (2017). Studying the old masters of nursing: a critical student experience for developing nursing identity. Nurse Educ. Pract. 26, 121–125. 10.1016/j.nepr.2017.06.010 28822955

[B25] Kyriazos T. A. (2018). Applied psychometrics: sample size and sample power considerations in factor analysis (EFA, CFA) and SEM in general. Psychology 9:2207. 10.4236/psych.2018.98126

[B26] Labrague L. J. De Los Santos J. A. A. Falguera C. C. Nwafor C. E. Galabay J. R. Rosales R. A.. (2020). Predictors of nurses' turnover intention at one and five years' time. Int. Nurs. Rev. 67, 191–198. 10.1111/inr.12581 32202329

[B27] Lent R. W. Brown S. D. Hackett G. (1994). Toward a unifying social cognitive theory of career and academic interest, choice, and performance. J. Vocational Beha. 45, 79–122. 10.1006/jvbe.1994.1027

[B28] Li X. Guo A. Zou H. (2022). Impact of the nurse-related information through social media use on undergraduate nursing students' professional identity in nursing: a mixed-methods study. Nurse. Educ. Pract. 65:103477. 10.1016/j.nepr.2022.103477 36327592

[B29] Lin S. Chen S. Tu Q. Xu X. Xie S. Yang B.. (2024). Barriers and facilitators to the formation of professional identity among nursing students: a four-year longitudinal qualitative study. Nurse Educ. Today 134:106087. 10.1016/j.nedt.2023.106087 38232627

[B30] Ling C. Yu S. (2023). The relationship between clinical work stress and anxiety in master's degree nursing students: the mediating role of psychological capital and social support. Medicine 102:e33997. 10.1097/MD.0000000000033997 37335666 PMC10256396

[B31] Liu Y. Aungsuroch Y. Gunawan J. Zeng D. (2021). Job stress, psychological capital, perceived social support, and occupational burnout among hospital nurses. J. Nurs. Scholarsh. 53, 511–518. 10.1111/jnu.12642 33646610

[B32] Liu Y. Han Y. Xiong L. Ma Q. Mei L. Chong M. C.. (2023). The mediating role of psychological capital in the relationship between job stress and professional identity in Chinese medical interns. Work 76, 1597–1604. 10.3233/WOR-230022 37393480

[B33] Luo W. Mao A. (2022). Impacts of COVID-19 pandemic on professional identity development of intern nursing students in China: a scoping review. PLoS One 17:e275387. 10.1371/journal.pone.0275387 36227891 PMC9560130

[B34] Luthans F. Avey J. B. Avolio B. J. Steven M. Norman G. M. C. (2006). Psychological capital development: toward a micro-intervention. J. Organ. Beha. 27, 387–393. 10.1002/job.373

[B35] Luthans F. Avey J. B. Patera J. L. (2008). Experimental analysis of a web-based training intervention to develop positive psychological capital. Acad. Manage. Learn. Educ. 7, 209–221. 10.5465/amle.2008.3271261824997007

[B36] Luthans F. Avolio B. J. Avey J. B. Norman S. M. (2007). Positive psychological capital: measurement and relationship with performance and satisfaction. Personnel Psycho. 60, 541–572. 10.1111/j.1744-6570.2007.00083.x

[B37] Luthans F. Luthans K. W. Luthans B. C. (2004). Positive psychological capital: beyond human and social capital. Bus. Horiz. 47, 45–50. 10.1016/j.bushor.2003.11.007

[B38] Luthans F. Youssef-Morgan C. M. Avolio B. J. (2015). Psychological Capital and Beyond. United States: Oxford University Press.

[B39] Mei X. X. Wang H. Y. Wu X. N. Wu J. Y. Lu Y. Z. Ye Z. J. (2022). Self-efficacy and professional identity among freshmen nursing students: a latent profile and moderated mediation analysis. Front. Psychol. 13:779986. 10.3389/fpsyg.2022.779986 35310284 PMC8927723

[B40] Mubarak N. Safdar S. Faiz S. Khan J. Jaafar M. (2021). Impact of public health education on undue fear of COVID-19 among nurses: the mediating role of psychological capital. Int. J. Ment. Health Nurs. 30, 544–552. 10.1111/inm.12819 33230850 PMC7753350

[B41] Qiu T. Liu C. Huang H. Yang S. Gu Z. Tian F.. (2019). The mediating role of psychological capital on the association between workplace violence and professional identity among Chinese doctors: a cross-sectional study. Psychol. Res. Behav. Manag. 12, 209–217. 10.2147/PRBM.S198443 31114405 PMC6474643

[B42] Ren Z. Zhang X. Li X. He M. Shi H. Zhao H.. (2021). Relationships of organisational justice, psychological capital and professional identity with job burnout among Chinese nurses: a cross-sectional study. J. Clin. Nurs. 30, 2912–2923. 10.1111/jocn.15797 33829587

[B43] Rodriguez-Garcia M. C. Gutierrez-Puertas L. Granados-Gamez G. Aguilera-Manrique G. Marquez-Hernandez V. V. (2021). The connection of the clinical learning environment and supervision of nursing students with student satisfaction and future intention to work in clinical placement hospitals. J. Clin. Nurs. 30, 986–994. 10.1111/jocn.15642 33432645

[B44] Sang N. Zhu Z. Wu L. Pei-li S. Wang L. Kan H.. (2022). The mediating effect of psychological resilience on empathy and professional identity of Chinese nursing students: a structural equation model analysis. J. Prof. Nurs. 43, 53–60. 10.1016/j.profnurs.2022.09.002 36496245

[B45] Sun L. Gao Y. Yang J. Wang X. Z. Y. (2016). The impact of professional identity on role stress in nursing students: a cross-sectional study. Int. J. Nurs. Stud. 63, 1–8. 10.1016/j.ijnurstu.2016.08.010 27565423

[B46] Tao H. Fan S. Zhao S. Xia Q. L. Y. Zeng L. Huang H. (2023). Mediating effects of transition shock and professional identity on the perception of a caring climate in hospitals and patient safety attitudes of nursing interns: a cross-sectional study. Nurse. Educ. Pract. 73:103836. 10.1016/j.nepr.2023.103836 37984162

[B47] Tyler D. McCallum R. S. (1998). Assessing the relationship between competence and job role and identity among direct service counseling psychologists. J. Psychoeduc. Assess. 16, 135–152. 10.1177/073428299801600203

[B48] Ugwu S. N. Ogbonnaya N. P. Chijioke V. C. Esievo J. N. (2023). Causes and effects of theory-practice gap during clinical practice: the lived experiences of baccalaureate nursing students. Int. J. Qual. Stud. Health Well-being 18:2164949. 10.1080/17482631.2023.2164949 36656608 PMC9858546

[B49] Vabo G. Slettebø Å. Fossum M. (2022). Nursing students' professional identity development: an integrative review. Nord. J. Nurs. Res. 42, 62–75. 10.1177/20571585211029857

[B50] Wei L. Zhou S. Hu S. Zhou Z. Chen J. (2021). Influences of nursing students' career planning, internship experience, and other factors on professional identity. Nurse Educ. Today 99:104781. 10.1016/j.nedt.2021.104781 33530029

[B51] WHO (2020). State of world's nursing. Available online at: https://www.who.int/publications/i/item/9789240003279 (accessed May 16, 2024).

[B52] Wu C. Palmer M. H. Sha K. (2020). Professional identity and its influencing factors of first-year post-associate degree baccalaureate nursing students: a cross-sectional study. Nurse Educ. Today 84:104227. 10.1016/j.nedt.2019.104227 31683135

[B53] Xia Y. Guo Q. Chen Q. Zeng L. Yi Q. Liu H.. (2023). Pathways from the clinical learning environment and ego identity to professional identity: a cross-sectional study. J. Prof. Nurs. 45, 29–34. 10.1016/j.profnurs.2023.01.006 36889891

[B54] Xiao S. Shi L. Lin H. Zhao S. Ou W. Zhang J.. (2022). The impact of psychological capital on turnover intention among Chinese nurses: a moderated mediation model. J. Nurs. Manag. 30, 3031–3040. 10.1111/jonm.13702 35661464

[B55] Xu Y. Zhang W. Wang J. Guo Z. Ma W. (2024). The effects of clinical learning environment and career adaptability on resilience: a mediating analysis based on a survey of nursing interns. J. Adv. Nurs. 1–10. 10.1111/jan.16144 38468419

[B56] Yang Q. Yang L. Yang C. Wu X. Chen Y. Yao P. (2023). Workplace violence against nursing interns and patient safety: the multiple mediation effect of professional identity and professional burnout. Nurs. Open 10, 3104–3112. 10.1002/nop2.1560 36567504 PMC10077394

[B57] Yao X. Yu L. Shen Y. Kang Z. Wang X. (2021). The role of self-efficacy in mediating between professional identity and self-reported competence among nursing students in the internship period: a quantitative study. Nurse. Educ. Pract. 57:103252. 10.1016/j.nepr.2021.103252 34781196

[B58] Yao X. Yu L. Shen Y. Kang Z. Wang X. (2023). Levels of psychological capital among nurses: a systematic review and meta-analysis. Int. Nurs. Rev. 70, 89–96. 10.1111/inr.12803 36205604

[B59] Zeng L. Chen Q. Fan S. Yi Q. An W. Liu H.. (2022). Factors influencing the professional identity of nursing interns: a cross-sectional study. BMC Nurs. 21:200. 10.1186/s12912-022-00983-2 35879704 PMC9310353

[B60] Zhang W. Liu L. Sun Y. Zhao F. (2022). Translation and reliability and validity of Chinese version Healthcare Education Micro Learning Environment Measure. Chin. J. Pract. Nurs. 38, 2801–2805. 10.3760/cma.j.cn211501-20220218-0044230704229

[B61] Zhang Y. Huang X. Xu S. Xu C. Feng X. Jin J. (2019). Can a one-on-one mentorship program reduce the turnover rate of new graduate nurses in China? A longitudinal study. Nurs. Educ. Pract. 40:102616. 10.1016/j.nepr.2019.08.010 31518894

[B62] Zhang Y. Juemin W. Fang Z. Zhang Y. Wong F. K. Y. FAAN F. (2017). Newly graduated nurses' intention to leave in their first year of practice in Shanghai: a longitudinal study. Nurs. Outlook 65, 202–211. 10.1016/j.outlook.2016.10.007 27939200

[B63] Zhao Y. Zhou Q. Li J. Luan J. Wang B. Zhao Y.. (2021). Influence of psychological stress and coping styles in the professional identity of undergraduate nursing students after the outbreak of COVID-19: a cross-sectional study in China. Nurs. Open 8, 3527–3537. 10.1002/nop2.902 33960736 PMC8242557

[B64] Zheng Y. Jiao J. Hao W. (2022). Stress levels of nursing students: a systematic review and meta-analysis. Medicine 101:e30547. 10.1097/MD.0000000000030547 36086725 PMC10980379

[B65] Zhong Y. Ma H. Zhang C. Jiang Q. Li J. Liao C.. (2024). Professional identity, job satisfaction, and turnover intention among Chinese novice nurses: a cross-sectional study. Medicine 103:e36903. 10.1097/MD.0000000000036903 38241583 PMC10798701

