# Supplementary material for: The relationship between psychological capital, stress, micro-learning environment, and professional identity in nursing interns: a structural equation modeling study
Source: Front Psychol. 2025 Mar 17;16:1458384. doi: 10.3389/fpsyg.2025.1458384 (PMC11955965; doi:10.3389/fpsyg.2025.1458384)
Supplement: Supplementary file 1 [file Table_1.docx]

**Supplementary TABLE 1**. Confirmatory factor analysis and reliability analysis (N=388)

|  | **Latent variables** | **Observed variables** | **Standard factor loading** | **SE** | **P** | **AVE** | **CR** |
| --- | --- | --- | --- | --- | --- | --- | --- |
| PsyCap | Self efficacy | PC1 | 0.776 |  |  | 0.652 | 0.9182 |
|  |  | PC2 | 0.805 | 0.06 | *** |  |  |
|  |  | PC3 | 0.783 | 0.065 | *** |  |  |
|  |  | PC4 | 0.828 | 0.052 | *** |  |  |
|  |  | PC5 | 0.855 | 0.056 | *** |  |  |
|  |  | PC6 | 0.795 | 0.053 | *** |  |  |
|  | Hope | PC7 | 0.798 |  |  | 0.6695 | 0.9235 |
|  |  | PC8 | 0.847 | 0.061 | *** |  |  |
|  |  | PC9 | 0.7 | 0.056 | *** |  |  |
|  |  | PC10 | 0.77 | 0.062 | *** |  |  |
|  |  | PC11 | 0.913 | 0.052 | *** |  |  |
|  |  | PC12 | 0.864 | 0.055 | *** |  |  |
|  | Resilience | PC13 | 0.795 |  |  | 0.6877 | 0.9167 |
|  |  | PC14 | 0.811 | 0.059 | *** |  |  |
|  |  | PC15 | 0.855 | 0.062 | *** |  |  |
|  |  | PC16 | 0.842 | 0.059 | *** |  |  |
|  |  | PC17 | 0.842 | 0.06 | *** |  |  |
|  | Optimism | PC18 | 0.916 |  |  | 0.8204 | 0.932 |
|  |  | PC19 | 0.911 | 0.035 | *** |  |  |
|  |  | PC20 | 0.89 | 0.035 | *** |  |  |
| Student Nurse Stress Index Scale | Academic load | S1 | 0.792 |  |  | 0.6334 | 0.896 |
|  |  | S2 | 0.831 | 0.056 | *** |  |  |
|  |  | S3 | 0.804 | 0.059 | *** |  |  |
|  |  | S8 | 0.829 | 0.064 | *** |  |  |
|  |  | S20 | 0.718 | 0.066 | *** |  |  |
|  | Clinical concerns | S13 | 0.796 |  |  | 0.6531 | 0.9186 |
|  |  | S14 | 0.766 | 0.059 | *** |  |  |
|  |  | S16 | 0.813 | 0.056 | *** |  |  |
|  |  | S17 | 0.823 | 0.055 | *** |  |  |
|  |  | S19 | 0.836 | 0.055 | *** |  |  |
|  |  | S18 | 0.813 | 0.055 | *** |  |  |
|  | Interface worries | S4 | 0.683 |  |  | 0.5614 | 0.8994 |
|  |  | S5 | 0.744 | 0.071 | *** |  |  |
|  |  | S6 | 0.762 | 0.074 | *** |  |  |
|  |  | S7 | 0.8 | 0.072 | *** |  |  |
|  |  | S15 | 0.733 | 0.073 | *** |  |  |
|  |  | S21 | 0.795 | 0.077 | *** |  |  |
|  |  | S22 | 0.721 | 0.082 | *** |  |  |
|  | Personal problems | S9 | 0.85 |  |  | 0.6356 | 0.8741 |
|  |  | S10 | 0.809 | 0.047 | *** |  |  |
|  |  | S11 | 0.701 | 0.049 | *** |  |  |
|  |  | S12 | 0.821 | 0.047 | *** |  |  |
| Healthcare Education Micro Learning Environment Measure | Staff attitudes and behaviors | H1 | 0.84 |  |  | 0.7149 | 0.9375 |
|  |  | H2 | 0.856 | 0.046 | *** |  |  |
|  |  | H3 | 0.87 | 0.044 | *** |  |  |
|  |  | H4 | 0.751 | 0.046 | *** |  |  |
|  |  | H5 | 0.856 | 0.046 | *** |  |  |
|  |  | H6 | 0.893 | 0.042 | *** |  |  |
|  | Teaching quality | H7 | 0.87 |  |  | 0.7006 | 0.9333 |
|  |  | H8 | 0.879 | 0.041 | *** |  |  |
|  |  | H9 | 0.877 | 0.04 | *** |  |  |
|  |  | H10 | 0.852 | 0.045 | *** |  |  |
|  |  | H11 | 0.734 | 0.056 | *** |  |  |
|  |  | H12 | 0.8 | 0.048 | *** |  |  |
| Professional identity | Professional identity | PI1 | 0.769 |  |  | 0.6256 | 0.9433 |
|  |  | PI2 | 0.822 | 0.068 | *** |  |  |
|  |  | PI3 | 0.846 | 0.069 | *** |  |  |
|  |  | PI4 | 0.83 | 0.086 | *** |  |  |
|  |  | PI5 | 0.668 | 0.102 | *** |  |  |
|  |  | PI6 | 0.772 | 0.093 | *** |  |  |
|  |  | PI7 | 0.773 | 0.077 | *** |  |  |
|  |  | PI8 | 0.856 | 0.071 | *** |  |  |
|  |  | PI9 | 0.815 | 0.075 | *** |  |  |
|  |  | PI10 | 0.74 | 0.071 | *** |  |  |

SE=standard error, CR=composite reliability, AVE=average variance extracted
